# The protocadherins, *PCDHB1 *and *PCDH7*, are regulated by MeCP2 in neuronal cells and brain tissues: implication for pathogenesis of Rett syndrome

**DOI:** 10.1186/1471-2202-12-81

**Published:** 2011-08-08

**Authors:** Kunio Miyake, Takae Hirasawa, Masaki Soutome, Masayuki Itoh, Yu-ichi Goto, Kazushi Endoh, Kenichiro Takahashi, Shinichi Kudo, Takayuki Nakagawa, Sana Yokoi, Takahiro Taira, Johji Inazawa, Takeo Kubota

**Affiliations:** 1 Department of Epigenetic Medicine, University of Yamanashi, Chuo, Yamanashi, Japan; 2 Department of Mental Retardation and Birth Defect Research, National Institute of Neuroscience, National Center of Neurology and Psychiatry, Kodaira, Tokyo, Japan; 3 Department of Pediatrics, School of Medicine, Showa University, Tokyo, Japan; 4 Hokkaido Institute of Public Health, Sapporo, Hokkaido, Japan; 5 Molecular Cytogenetics, MRI, Tokyo Medical Dental University, Tokyo, Japan; 6 Department of Molecular Cellular Biology, University of Yamanashi, Chuo, Yamanashi, Japan

## Abstract

**Background:**

Rett syndrome is a neurodevelopmental and autistic disease caused by mutations of *Methyl-CpG-binding protein 2 *(*MECP2*) gene. MeCP2 protein is mainly expressed in neurons and binds to methylated gene promoters to suppress their expression, indicating that Rett syndrome is caused by the deregulation of target genes in neurons. However, it is likely that there are more unidentified neuronal MeCP2-targets associated with the neurological features of RTT.

**Results:**

Using a genome-microarray approach, we found 22 genomic regions that contain sites potentially regulated by MeCP2 based on the features of MeCP2 binding, DNA methylation, and repressive histone modification in human cell lines. Within these regions, Chromatin immunoprecipitation (ChIP) analysis revealed that MeCP2 binds to the upstream regions of the protocadherin genes *PCDHB1 *and *PCDH7 *in human neuroblastoma SH-SY5Y cells. PCDHB1 and PCDH7 promoter activities were down-regulated by MeCP2, but not by MBD-deleted MeCP2. These gene expression were up-regulated following MeCP2 reduction with siRNA in SH-SY5Y cells and in the brains of *Mecp2*-null mice. Furthermore, *PCDHB1 *was up-regulated in postmortem brains from Rett syndrome patients.

**Conclusions:**

We identified MeCP2 target genes that encode neuronal adhesion molecules using ChIP-on-BAC array approach. Since these protocadherin genes are generally essential for brain development, aberrant regulation of these molecules may contribute to the pathogenesis of the neurological features observed in Rett syndrome.

## Background

Methyl-CpG-binding protein 2 (MeCP2) is one of the proteins associated with epigenetic regulation, and mutations of this gene have been identified in the majority of patients with a severe neurodevelopmental disorder, Rett syndrome (RTT), characterized by seizures, ataxic gait, language dysfunction, and autistic behavior [1,2]. *Mecp2*-null mice exhibit neurological abnormalities strikingly similar to those of RTT, supporting the hypothesis that classical RTT is due to a loss of MeCP2 function [3,4], and that MeCP2 is essential for neuronal development, maturation, synaptic activity, learning and memory [5-7].

MeCP2 has been thought to be a transcriptional repressor that acts by binding to a number of methylated-CpG dinucleotides in the mammalian genome. However, its deficiency does not result in the significant deregulation of the expression of a subset of genes as determined by a comparative expression microarray analyses between *Mecp2*-null mice and wild-type mice [8], but induces global changes in neuronal chromatin structure [9]. These findings indicate that MeCP2 may be a global gene silencer. Furthermore, MeCP2 deficiency affects the expression levels of a large number of genes as determined by a comparative expression microarray analyses between *Mecp2*-knock-in mice and *Mecp2*-duplication mice [10], indicating that MeCP2 target genes are numerous. However, it is still worthwhile to identify MeCP2-target genes which that are centrally involved in RTT pathogenesis, since MeCP2 functions cell-autonomously in neuronal maturation and dendritic arborization and discrete subsets of genes regulated by MeCP2 may be essential for mature neuronal function [11].

So far several genes associated with neuronal function have been reported as MeCP2 targets, such as brain-derived neurotrophic factor (*BDNF*) [12,13], glucocorticoid-regulated genes [14], interleukin-1 receptor-associated kinase 1 (*IRAK 1*) [15], insulin-growth factor binding protein 3 (*IGFBP3*) [16], a transmembrane modulator of Na^+^, K^+^-ATPase activity (*FXYD1*) [17], and cyclin-dependent kinase-like 5 (*CDKL5*) [18]. However, it is likely that there are more unidentified neuronal MeCP2-targets associated with the neurological features of RTT.

In this study, we used a genome-microarray based approach [19,20] rather than a standard expression-microarray approach, to identify genomic regions that are epigenetically regulated by MeCP2.

## Results

### Screening for BACs containing MeCP2 binding sites with epigenetic modification

We assumed that MeCP2 was bound to multiple sites in human genome. In order to clarify these sites, we first performed ChIP on chip assay using our in-house BAC array ("ChIP on BAC array" assay) with an anti-MeCP2 antibody in human oral cancer cell lines (ZA, KOSC2, HSC5, NA). As a result, we obtained 846 "positive" BAC clones, which were suggestive of having MeCP2 binding sites, out of the 4,500 clones on the array (data not shown). We next screened BAC clones encompassing hypermethylation site(s) and repressive histone modification sites based on DNA methylation using the BAMCA and ChIP-on-BAC array assay with an anti-histone H3K9-2Me antibody in the same cell lines [19,20]. We identified 22 "triple positive" BAC out of the 846 "MeCP2 positive" BAC clones, which contain MeCP2-binding site(s), hypermethylation site(s), and repressive histone modification site(s). Although "triple positive" did not necessary mean these three epigenetic modifications existed at the same genomic site in a genomic region (~300 kb) in a BAC, we considered that the genomic regions in these 22 BAC clones potentially contained the site(s) regulated by MeCP2. At this step, we used oral cancer cell lines because they had previously screened to identify MeCP2 targets in carcinogenesis (manuscript in preparation), and used this screening data in this study.

### Search for Neuronal Genes

We next searched for neuronal genes in the genomic regions within these 22 BAC clones using a genome database (NCBI MAPVIEW), and identified the following four genes: *APBB3 *(amyloid beta precursor protein-binding, family B, member 3) and *SRA1 *(steroid receptor RNA activator 1) in BAC RP11-115I4 (located at chromosome 5q31.3), *PCDHB1 *(protocadherin beta 1) in BAC RP11-79K4 (5q31.3), and *PCDH7 *(protocadherin 7; brain-heart protocadherin) in RP11-205N12 (4p15.1). Among these genes, the consensus MeCP2-binding sequence with A/T bases [A/T≥4] within 1-3 or 6-9 base pairs from a CpG di-nucleotide [21] was identified in the 5' flanking region (within 1 kb region from the transcriptional start site) in *APBP3, PCDHB1 *and *PCDH7*, but not in *SRA1 *(data not shown).

### MeCP2-binding status in the upstream regions of the neuronal genes in SH-SY5Y human neuroblastoma cells

Since the observed epigenetic alterations were found in oral cancer cell lines, we confirmed whether MeCP2 was bound to the *APBP3, PCDHB1 *and *PCDH7 *genes using the ChIP-PCR assay in human neuroblastoma SH-SY5Y cells as neuronal origin cells.

**Figure 1:**
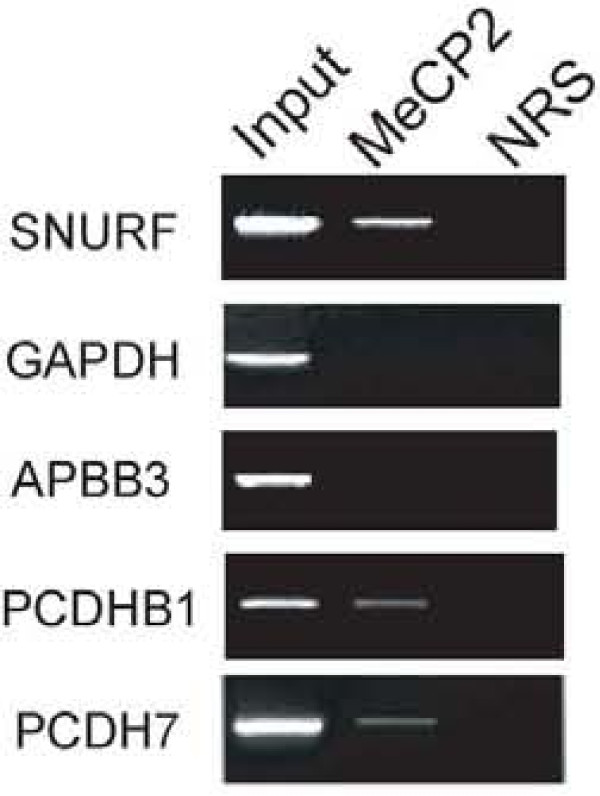
**MeCP2 binds to the promoter region of two target genes in SH-SY5Y cells**. Immunoprecipitation (IP) was performed using an anti-MeCP2 antibody or normal rabbit serum (NRS) as negative control. Equal amounts of precleared chromatin were processed without IP as total input control. The purified DNA was amplified by PCR using primers located within the 1.0 kb upstream genomic regions from the transcriptional start sites of the *APBB3, PCDHB1, or PCDH7 *genes. *SNURF/SNRPN *was used as a positive control for a promoter previously demonstrated to bind MeCP2. GAPDH was used as a negative control.

Within the 5' flanking regions of these genes, we found that MeCP2 was bound to the regions of *PCDH7, PCDHB1 *genes, but not to the *APBP3 *gene (Figure 1). In this assay, we confirmed that MeCP2 was bound to the promoter region of *SNURF/SNRPN *gene (a known MeCP2 target site) but not bound to the promoter region of the GAPDH gene (a known non-MeCP2 target site) in SH-SY5Y cells [22].

### Methylation status in the upstream regions of the two neuronal genes in SH-SY5Y human neuroblastoma cells

**Figure 2:**
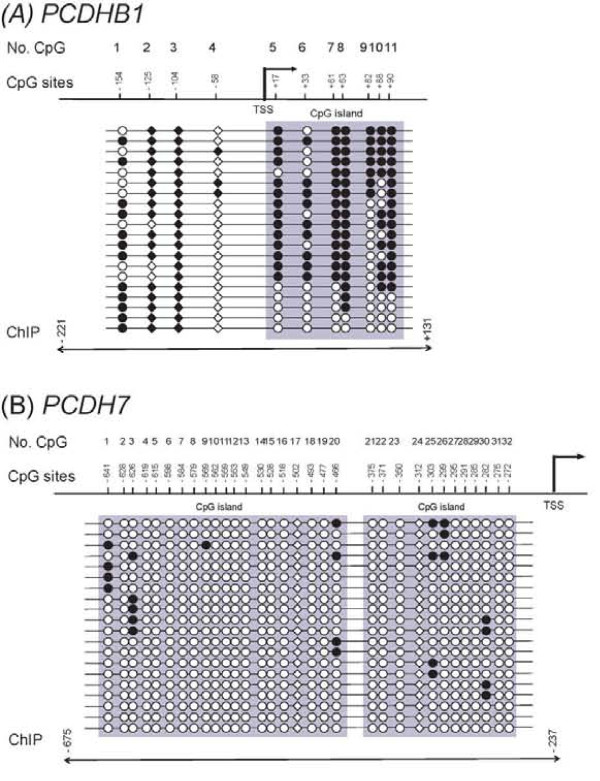
**Mapping the methylation status of the *PCDHB1 *(A) and *PCDH7 *(B) regions in SH-SY5Y cells by bisulfite genomic DNA sequencing**. The top diagram depicts the 5' flanking region of each gene with its transcription start site. CpG island regions are shown as gray areas. Methylated-CpG sites are shown as closed circles and unmethylated CpG sites as open circles. The triangles represent the CpG sites with A/T bases ([A/T]_>4_) located 1-3 or 6-9 base pairs from the CpG sites, indicating the putative MeCP2-binding sequences. Arrows at the bottom indicate the region that was amplified for ChIP-PCR. (A) 5' flanking region of the *PCDHB1 *gene. (B) 5' flanking region of the *PCDH7 *gene.

We examined the methylation status in the upstream regions of the *PCDHB1 *and *PCDH7 *genes, in order to determine whether the CpG sites were hypermethylated for MeCP2 binding (Figure 2A, B). The *PCDHB1 *up-stream region was highly methylated as expected. However, the *PCDH7 *up-stream region was unexpectedly less methylated, and the result was nonetheless consistent with a recent report, in which MeCP2 not only binds to highly methylated regions, but also binds to less methylated regions [23].

### Suppression of *PCDHB1 *and *PCDH7 *genes by MeCP2 in SH-SY5Y human neuroblastoma cells

**Figure 3:**
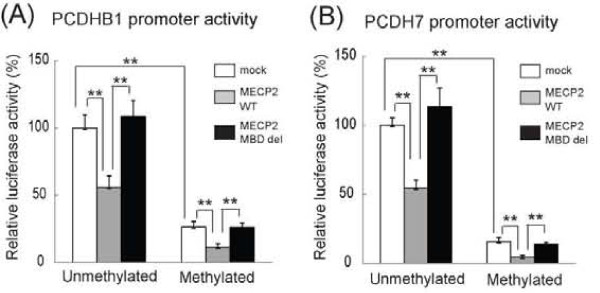
**Effects of wild-type MECP2 or MBD-deleted mutations on the transcriptional activity of the methylated or unmethylated PCDHB1 and PCDH7 promoter fragments**. SH-SY5Y cells were transfected with the methylated or unmethylated PCDHB1-luc (A), PCDH7-luc (B) as a reporter vector. As an effector, an *MECP2*-expression vector was co-transfected. After 48 h, the transfectants were lysed and assayed for luciferase activity. *PCDHB1 *and *PCDH7 *promoter transcriptional activities are repressed by wild-type MeCP2 (gray), but not by MBD-deleted mutant (black) in SH-SY5Y cells. Luciferase reporter activity in each sample was normalized according to the beta-galactosidase activity measured in the same sample. The luciferase activity of the cells transfected with the reporter vector was taken as 100%. All results are shown as the mean ± SEM of three replicates.

To examine whether the expression of the *PCDHB1 *and *PCDH7 *genes was controlled by MeCP2, we evaluated the effects of wild-type or MBD-deleted MECP2 on either unmethylated or methylated promoter by the luciferase assay in SH-SY5Y cells. Methylation status of these constructs was confirmed by bisulfite sequencing (data not shown). Luciferase fusion plasmids containing 1.5 kb of upstream sequences of the *PCDHB1 *or *PCDH7 *transcription start site were methylated by methylase SssI *in vitro*. These luciferase reporter plasmids were co-transfected in combination with the *MECP2*-expressing plasmid into SH-SY5Y cells. The transcriptional activity of the unmethylated *PCDHB1 *promoter was suppressed by wild-type MeCP2, but not MBD-deleted mutant MeCP2 (Figure 3A). Likewise, the transcriptional activity of the methylated *PCDHB1 *promoter was suppressed by wild-type MeCP2, but not MBD-deleted mutant MeCP2 (Figure 3A). Approximately 70% reduction in promoter activity was found in methylated promoters, compared with unmethylated promoters, by mock (no MeCP2), indicating that endogenous MeCP2 preferentially suppresses *PCDHB1 *methylated promoter (Figure 3A). Similar to *PCDHB1*, the transcriptional activity of both the unmethylated and methylated PCDH7 promoter was suppressed by wild-type MeCP2, but not MBD-deleted mutant MeCP2 (Figure 3B). We did not find down-regulation of the SV2 promoter region by wild-type MeCP2, suggesting that the *SV2 *gene is not a MeCP2-target gene (data not shown). Taken together, these results indicate that not only methylated but also unmethylated promoter of the *PCDHB1 *and *PCDH7 *genes are regulated by MeCP2 in SH-SY5Y cells.

### Knock-down effect on the two neuronal genes with MECP2-siRNA in SH-SY5Y human neuroblastoma cells

**Figure 4:**
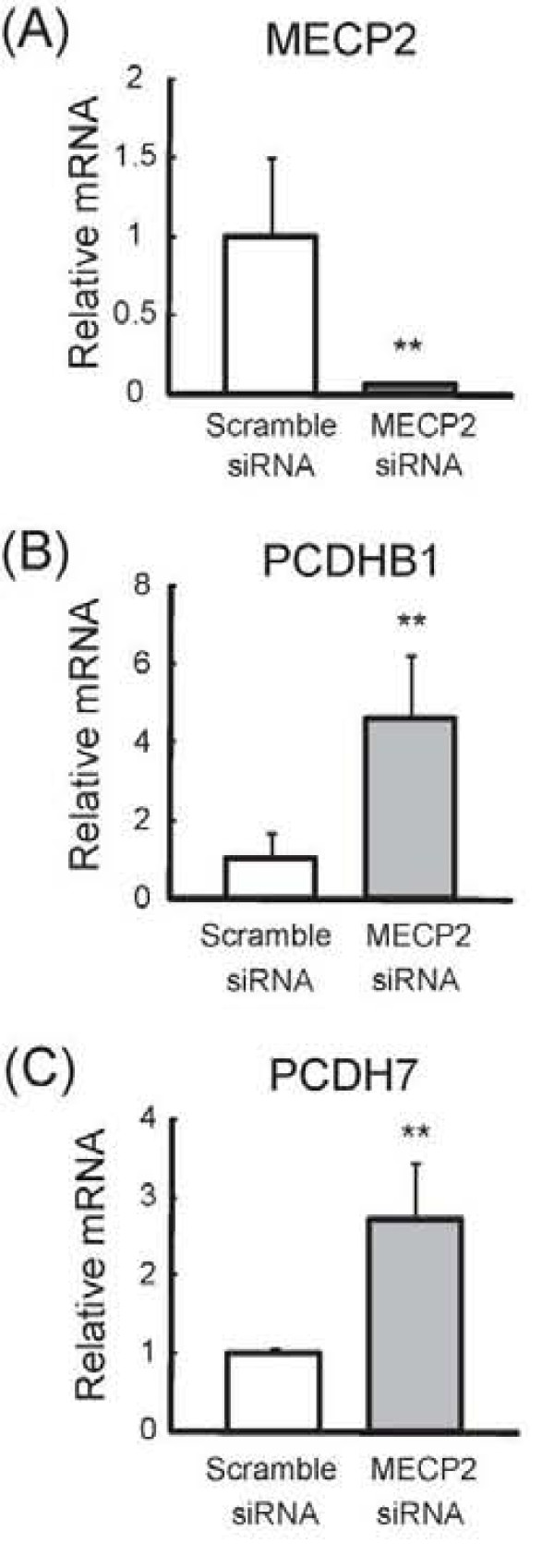
**Expression of *PCDHB1 *and *PCDH7 *by RNAi-mediated knockdown of MECP2 in SH-SY5Y cells**. SH-SY5Y cells were transfected with *MECP2*-siRNA or Scramble siRNA (Control) for 24 h. The expression level of *MECP2 *(A), *PCDHB1 *(B) and *PCDH7 *(C) were examined by qRT-PCR and normalized using the expression level of GAPDH. All results are shown as the mean ± SEM of three replicates with the mean Control normalized to 1.0.

If *PCDHB1 *and *PCDH7 *genes are controlled by MeCP2, their expression from these genes should be increased under the MeCP2 deficient condition. To assess this, we performed knockdown experiments using *MECP2*-siRNA. We first confirmed the transfection efficiency of siRNA in SH-SY5Y cells, and the fluorescent labeled siRNA showed that the efficiency of siRNA delivery was approximately 50~60%. As a result, the expression level of *MECP2 *mRNA decreased by 85% by following treatment with *MECP2*-siRNA compared with scramble-siRNA (Figure 4A). Under this condition, the expression levels of *PCDHB1 *and *PCDH7 *mRNA were significantly increased by *MECP2*-siRNA compared with scramble-siRNA (4.9- and 2.7-fold, respectively) (Figure 4B, C).

### Expression levels of the two neuronal genes in Mecp2-null mice and RTT brain tissue samples

**Figure 5:**
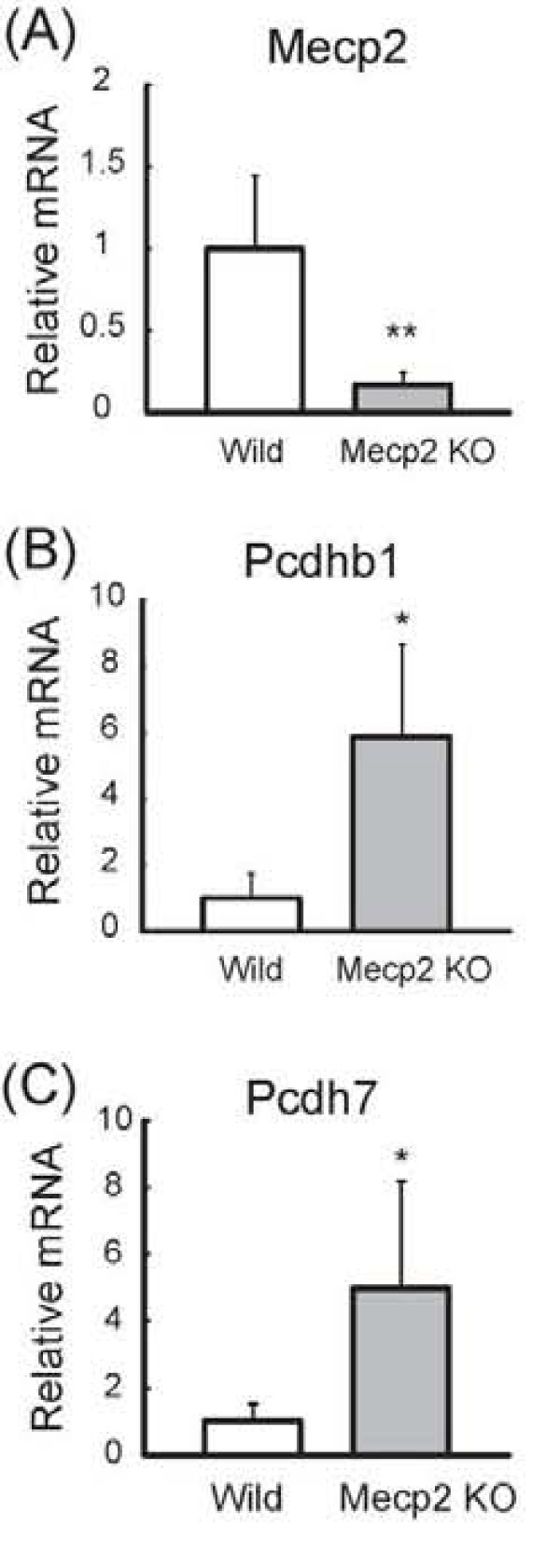
**Comparison of *Pcdhb1 *and *Pcdh7 *expression in cortical tissues from wild-type and *Mecp2*-null mice**. Total RNA was extracted from the *Mecp2*-null and wild-type mice at postnatal day 14 (P14) and cDNA was synthesized with random primers. The expression levels of Mecp2 (A), Pcdhb1 (B) and Pcdh7 (C) were examined by qRT-PCR and normalized using the expression level of Gapdh. All results are shown as the mean ± SEM of three replicates with the mean Control normalized to 1.0.

We examined the expression of the two genes in brain tissue samples from Mecp2-null mice (Mecp2 ^tm1.1 Bird^) [4]. We compared the mRNA expression level in the frontal cortex of 14-day-old *Mecp2*-null mice with those of wild-type male mice. As a result, we found that Pcdhb1 and Pcdh7 mRNA were significantly increased in *Mecp2*-null mice compared with wild-type mice (5.0- and 5.9-fold respectively) (Figure 5).

**Figure 6:**
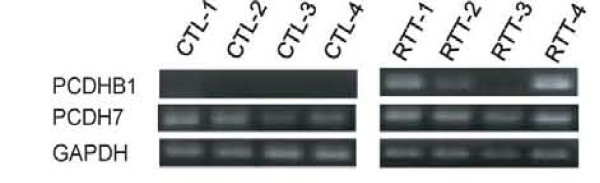
**Comparison of *PCDHB1 *and *PCDH7 *expression in the cortical tissues from four control individuals and four RTT patients**. Total RNA was extracted from postmortem brain cortical tissue in controls (CTR-1~4) and RTT patients (RTT-1~4). The expression levels of *PCDHB1, PCDH7 *and *GAPDH *were examined by RT-PCR.

We further investigated the expression of these genes in postmortem RTT brain tissues. As a result, the aberrant expression of the *PCDHB1 *gene was found in three of four RTT patients (RTT-1, RTT-2 and RTT-4) compared with control individuals (Figure 6). However, there was no apparent difference in the expression of *PCDH7 *gene in the brain tissue of controls and RTT individuals.

## Discussion

It had been thought that the causative gene for RTT should encode a synapse-associated molecule based on its pathogenesis. However, the gene in which most RTT patients have mutations does not encode a synapse molecule, but encodes an epigenetic regulation protein. This raises the question about which synaptic molecules are regulated by MeCP2, and directly contribute to its neuropathogenesis. To address this question, several attempts to identify MeCP2 target genes have been performed. The initial study using a expression microarray demonstrated that subtle expression changes occur in the brain of *Mecp2*-null mice [8], indicating that the accumulation of subtle changes affect brain function and that brains are less tolerant of background transcriptional noise than other organs [24]. To date, several neuronal molecules regulated by MeCP2, such as *BDNF, IGFBP3 *and CRMP1 have been identified by candidate gene approaches or MeCP2 target screenings using expression microarrays [12-16]. To our knowledge, this is the first attempt to identify MeCP2 target genes using ChIP-on-BAC array approach using a genome microarray. We identified two genes PCDHB1 and PCDH7 that encode molecules associated with neuronal function.

However, we did not detect the previously reported MeCP2 target genes probably because our in-house array only covers one third of the human genome and the genomic loci of previously identified genes might not be located within the overlapping regions with MeCP2 binding, DNA methylation and repressive histone modification, although the reported genes are located at sites where MeCP2 is bound. A newly-developed ChIP-sequencing approach using a next-generation sequencer, which is a more quantitative method to assess methylation [25], will shed light on the identification of new MeCP2 target genes.

It has been thought that MeCP2 represses transcription by binding specifically to methylated DNA. However, it was recently reported that MeCP2 is also bound to unmethylated DNA [26,27]. In this context, our data supported this notion, because MeCP2 repressed transcriptional activity of *PCDHB1 *and *PCDH7 *genes either via methylated or unmethylated promoter constructs. However, the transcriptional activity was more effectively repressed via the methylated promoter constructs than via the unmethylated promoter constructs, which are consist with a report showing that the affinity of MeCP2 for methylated DNA is ~3-fold greater than unmethylated DNA [26].

The protocadherins comprise the largest subfamily of the cadherin superfamily and are predominantly expressed in the nervous system [28]. They are divided into two groups (the clustered and nonclustered Pcdh families) based on their genomic structure. PCDHB1 and PCDH7 are belonged to the clusterd and nonclustered families, respectively. Genome association studies have shown that single-nucleotide polymorphisms and deletions in Pcdh genes, such as PCDH10, PCDH11Y and PCDH12, are associated with bipolar disorder, schizophrenia and autism, respectively [29-33].

The clustered Pcdh family is subdivided into three distinct gene groups in mammals (Pcdh-α, Pcdh-β, and Pcdh-γ). Pcdh-α expression is down regulated by myelination during neuronal maturation [34,35], and Pcdh-β, namely *PCDHB16*, is expressed in dendritic spines and plays an important role in synaptogenesis [36]. Since the expression of PCDHB1 is not detectable in normal brains during development [37], the presence of *PCDHB1 *in the brains of RTT patients and the up-regulation of *PCDHB1 *in *Mecp2*-null mice may be associated with the neurological findings in RTT brains, such as decreased neuronal size, increased cell density and reduced dendritic arborization [5,37-39]. Furthermore, since our results indicate that PCDHB1 is epigenetically regulated by MeCP2, the *Pcdh*-β gene cluster may be epigenetically regulated similar to the *Pcdh-*α gene cluster in which epigenetic regulation produces isoforms in neurons [40].

PCDH7 is predominantly expressed in the somatosensory and visual cortices in the cerebral cortex, external granule cell layer in the cerebellar cortex, and the brainstem starting from embryonic day 17, and PCDH7 exhibits a critical period for the establishment of specific synaptic connections [41,42]. PCDH7 is also expressed in the ganglion cell layer of the retina [43], and its over-expression leads to a morphological change and Ca^2+^-dependent cell adhesion in mouse fibroblast L cells [44]. Therefore, the up-regulation of *PCDH7*, observed in the brains of *Mecp2*-null mice and neuroblastoma cells following *MECP2*-siRNA treatment, could potentially alter synaptic connections. However, no up-regulation was found in the brain tissues of RTT patients, and this may be due to the area of the brain examined (prefrontal cortex). Another finding in our study, in which the upstream region of *PCDH7 *was unexpectedly unmethylated despite its transcriptional respression by MeCP2 binding to its promoter, was consistent with the recent report that MeCP2 can bind to less methylated regions of genes and repress their expression [45].

Several lines of evidence suggest that MeCP2 acts as (1) a promoter of neuronal differentiation [46,47], (2) an effecter of dendritic arborization [3,5,38,39], (3) a modulator of synapses in postmitotic neurons), (4) an essential factor for the maturation of NMDA receptors [48] and (5) a controller of the balance between excitatory and inhibitory synaptic transmission through the maintenance of density between glutamate and GABA receptors [49-54]. Here we show that MeCP2 also regulates protocadherins, including PCDH7 that is potentially associated with synaptogenesis. Therefore, our findings may help to clarify the pathogenesis of RTT with synaptic dysfunction.

## Conclusions

In this study we identified two novel direct MeCP2 target genes by ChIP and reporter assay. Expression of PCDHB1 and PCDH7 were regulated by MeCP2 in human neuroblastoma cells and brain tissue. On the basis of the previous findings of the nature of protocadherins, dysregulation of these molecules are potentially associated with the neuronal and synaptic dysfunction observed in the brains of RTT patients.

## Methods

### Cell culture

Four human oral cancer cell lines (ZA, KOSC2, HSC5, NA) and a human neuroblastoma cell line (SH-SY5Y) were maintained in Dulbecco's modified Eagle's medium (DMEM) (Sigma Aldrich, St. Louis, MO) supplemented with 10% fetal calf serum at 37°C in a 5% humidified atmosphere.

### Bacterial artificial chromosome screening

Bacterial artificial chromosomes (BACs), which contain MeCP2 binding site(s), and repressive histone modification (H3K9) site(s), were screened using a ChIP-on-BAC array. Briefly, Chromatin Immunoprecipitation (ChIP) samples were prepared from the four cell lines using an anti-C-terminal anti-MeCP2 antibody and an anti-histone H3K9-2Me antibody (Abcam, Cambridge, UK) as described below. The antibody-enriched immunoprecipitate and total input control were amplified by adaptor PCR and labeled with Cy3-dCTP and Cy5-dCTP, respectively. Labeled test and control PCR products were co-hybridized to our in-house BAC-array (MCG Whole Genome Array-4500). Hybridizations were carried out as described elsewhere [55]. Arrays were scanned with a GenePix 4000 B (Axon Instruments, Foster City, CA) and analyzed using GenePix Pro 4.1 software (Axon Instruments).

BACs, which contains DNA methylated region(s), were screened using BAC array-based methylated CpG island amplification (BAMCA) [19,20] analyses of the four cell lines (ZA, KOSC2, HSC5, and NA). Briefly, the preparation of DNA probes for screening of methylated regions was carried out by the Methylated CpG island Amplification (MCA) method [56]. Five-microgram aliquots of test DNA (extracted from the four cell lines) were first digested with 100 units of a methylation-sensitive restriction enzyme *Sma*I and subsequently with 20 U of methylation-insensitive *Xma*I. Adaptors were ligated to the *Xma*I-digested sticky ends and PCRs were performed using an adaptor primer and Cy3-dCTP for labeling. Control DNA (extracted from primary cultured cells of normal oral mucosa) was treated in the same manner except that they were labeled with Cy5-dCTP. Hybridization and array scanning were performed described above.

In either assay (ChIP-on-BAC array with MeCP2 antibody, ChIP-on-BAC array with histone H3K9 antibody, and BAMCA), if a BAC demonstrated a Cy3/Cy5 intensity ratio more than 1, it was recognized as a "positive" BAC.

### Chromatin immunoprecipitation

Immunoprecipitation and reverse crosslinking were performed using a ChIP Assay Kit (Millipore, Billerica, MA) with C-terminal anti-MeCP2 antibody (Abcam) or normal rabbit serum (NRS) (Wako, Osaka, Japan) as a negative control according to the manufacturer's instructions. Equal amounts of precleared chromatin were processed without IP as total input control. Immunoprecipitates collected by centrifugation were washed, then digested with 50 mg/ml DNase free RNase A for 30 min at 37°C, followed by SDS/proteinase K digestion and subjected to phenol/chloroform extraction before ethanol precipitation with glycogen. One twentieth of the DNA from each IP reaction was PCR amplified in reactions containing 2.5 U of AmpliTaq GOLD (Applied Biosystems, Norwalk, CT), with buffer II, dNTP mix (2.5 mM each), and 0.2 mM primers of either *GAPDH*-F (5' - CCAATCTCAGTCCCTTCCCCC -3') and *GAPDH*-R (5' - GTTTCTCTCCGCCCGTCTTC -3') specific to the *GAPDH *promoter region (Fulmer-Smentek et al., 2001), *SNURF*-F (5' - ACTGCCATAGCCTCCTCGCCTC - 3' and *SNURF*-R (5' -CTTGCTGTTGTGCCGTTCTGCC - 3') specific to the *SNURF/SNRPN *promoter region within the 15q11-13 imprinting control region (Thatcher et al., 2005), *APBB3*-F (5' - CCTGGATGGGCTTTACCTCT - 3') and *APBB3*-R (5' - AACAGTGTGGAGTGGTGTGG - 3') specific to the *APBB3 *upstream region, *PCDHB1*-F (5' - TCAGTGGCTCCAGACAGCTA - 3') and *PCDHB1*-R (5' - TGCCACTGAATAGCGGATAG - 3') specific to the *PCDHB1*- upstream region, or *PCDH7*-F (5' - GACAAGCCTGATCCGTGAG - 3') and *PCDH7*-R (5' - GCAGGGAACTCAAGCTGAAC - 3') specific to the *PCDH7*- upstream region, using one cycle of 95°C for 10 min, 33-35 cycles of 95°C for 30 s, 55 or 60°C for 30 s, 72°C for 30 s, with a final cycle of 72°C for 7 min. PCR products were resolved by agarose gel electrophoresis and stained with ethidium bromide. Primers were designed within 1.0 kb upstream genomic regions from the transcriptional start sites of either the *APBB3, PCDHB1, or PCDH7 *gene, which contain CpG islands that fulfilled our criteria (>100 base pairs; % of C or G >50%; Observed CG / Expected CG > 0.6), and the putative MeCP2-binding sequences with A/T bases ([A/T]_>4_) located 1-3 base pairs or 6-9 base pairs from the CG site [21].

### DNA methylation analyses

Genomic DNA was extracted from SH-SY5Y cells using a DNeasy Blood and Tissue Kit (Qiagen, Hilden, Germany), and was subjected to sodium bisulfite modification with an EpiTect bisulfite kit (Qiagen). Modified DNA was amplified by PCR with the primers *PCDHB1*-BF (5' - TTTGAAAGGGAATTAATAGGTGAGTTTG - 3') and *PCDHB1*-BR (5' - TCCCCCACAAATATACACAAAAAAATA - 3') specific to the *PCDHB1*- upstream region, and *PCDH7*-BF (5' - TATTTAGTAGTAATTATTATTTTGGGTAAT - 3') and *PCDH7*-BR (5' - ATTCAAAAATAAACAAACCAAACTC - 3') specific to the *PCDH7*-upstream region, using one cycle of 95°C for 10 min, 33-35 cycles of 95°C for 30 s, 55 or 60°C for 30 s, 72°C for 30 s, with a final cycle of 72°C for 7 min. The primers used in this analysis were located in the same regions as those used in the ChIP analysis described above. Each PCR product was cloned into a p*PCR4 *vector using a TOPO TA Cloning Kit (Invitrogen, Carlsbad, CA) and sequenced.

### MECP2-expression vectors

To make an *MECP2*-expression plasmid, an *MECP2 *cDNA (I.M.A.G.E #03956518, Geneservice, Cambridge, UK) was inserted into the TA cloning vector (Invitrogen), and then an *EcoRI-XhoI *fragment of the *MECP2 *cDNA, created with primers containing either an *EcoRI *or *XhoI *site, was inserted into pc*DNA3 *(Invitrogen). *MECP2 *cDNA lacking the methyl-binding domain (MBD) was made by PCR amplification using KOD-Plus-Mutagenesis Kit (Toyobo, Osaka, Japan). The primers of MECP2-MBDdel-f (5' - GGGAGCCCCTCCCGGCGAGAGCAG - 3') and MECP2-MBDdel-r (5' - AGCTTCCGGCACAGCCGGGGCGGAG - 3'), located at both flanking sites of MBD in opposite directions to each other, were used for PCR with pcDNA3 containing the normal *MECP2 *cDNA sequence as a template DNA.

### In vitro methylation of reporter plasmid

In vitro methylation of reporter plasmids was carried out as reported previously [57]. Briefly, to make a reporter plasmid, we amplified approximately 1.5 kb of the upstream genomic regions from the transcription start site with primers *PCDHB1*-RF (5' - GGGGTACCAAGAGGAAAATGAGAGCACACC - 3') and *PCDHB1*-RR (5' - GGAAGCTTAGCCAACTGTTGCGGATATACT - 3') specific to the *PCDHB1 *upstream region, and *PCDH7-*RF (5' - GGGGTACCACACTTCCATCCAACGGGCATCTAC - 3') and *PCDH7*-RR (5' - GGAAGCTTCTCTGCGCAAGGTCATTAGTCACG - 3') specific to the *PCDH7 *upstream region, and these were fused upstream of the firefly luciferase gene in the pGL3-Basic vector (Promega). Whole PCDHB1 and PCDH7 reporter plasmids were methylated using SssI methylase, which methylates all cytosine residues within the double-stranded dinucleotide recognition sequence 5'-CG-3'.

Each plasmid was digested with KpnI/HindIII and then incubated with 3 U/μg of M.*Sss*I. The same plasmid was mock methylated in the absence of M.*Sss*I. Methylated and mock-methylated plasmids were religated back to KpnI/HindIII-restricted pGL3-Basic vector. The extent of methylation was determined by bisulfite sequence.

### Luciferase assay

One microgram of the *MECP2 *expression plasmid, 3 μg of the reporter plasmid and 1 μg of SV2-β-gal, a β-galactosidase expression vector, were transfected into SH-SY5Y cells (approximately 80% confluent) in a 6-cm dish using the FuGENE HD transfection reagent (Roche) as described by the manufacturer. Two days after transfection, whole cell extracts were prepared by the addition of the Triton X-100-containing solution from a Pica gene kit (Wako) to the cells. Approximately a one-fifth volume of the extract was used for the β-galactosidase assay to normalize the transfection efficiency as described previously [58], and luciferase activity was determined using the Pica gene kit and a Luminometer, Lumat LB9501 (EG & G Berthold, Berlin, Germany). The same experiments were repeated five times.

### RNAi knockdown

Twenty nanomolar MECP2-siRNA (SI02664893, Qiagen) were transfected into SH-SY5Y cells using the Hiperfect transfection Reagent (Qiagen). To determine the efficiency of siRNA delivery into the cells and the efficiency of the reduction of gene expression, we used 20 nM of an Alexa 488 fluorescence labeled control siRNA (1027284, Qiagen) and siRNA for the *MARK1 *gene (SI03650367, Qiagen). We also used a scrambled siRNA (1027284, Qiagen) as a negative control. At 24 h after transfection, cells were harvested for use in further experiments.

### Mouse and human tissue samples

B6.129P2 (C)-*Mecp2 *^tm1.1Bird/J ^mice lacking exons 3 and 4 were obtained from the Jackson Laboratory (Bar Harbor, ME). *Mecp2*-null mice and wild-type male littermates, as controls, were used at postnatal day 14 (P14). All animal experiments were approved by the University of Yamanashi Animal Care and Use Committee. Postmortem brain (cortex) samples from individuals were obtained with informed consent and postmortem brain samples from RTT patients were obtained from the Harvard Brain Tissue Bank, USA. The profile of each individual is shown in Table 1.

**Table 1:** Characteristics of the RTT patient and control brain samples used in this study

	Age	Sex	Diagnosis	Mutation	Cause of death
CTL-1	23 y	M	Duchanne muscular dystrophy	-	Pulmonary infection

CTL-2	11 y	M	healthy boy	-	Cardiac infarction

CTL-3	69 y	M	ALS	-	Pneumonia

CTL-4	37 y	M	CP (Athetosis)	-	Hepatocellular carcinoma

RTT-1	8 y	F	Rett syndrome	R255X	Drowning

RTT-2	24 y	F	Rett syndrome	R255X	Respiratory failure

RTT-3	10 y	F	Rett syndrome	R270X	unknown

RTT-4	20 y	F	Rett syndrome	N.D.	unknown

### RNA extraction and quantitative reverse transcription PCR

Total RNA was extracted from the mouse and human brain (cerebral cortices) samples using an RNeasy mini kit (Qiagen). Total RNA was reverse-transcribed with random primers and Ominscript reverse transcriptase (Qiagen) according to the manufacturer's instructions. One tenth of the reaction was used in the PCR amplification. Gene expression was measured by quantitative reverse transcription PCR (qRT-PCR) on an ABI Prism 7500 with a QuantiTect SYBR Green PCR kits (Qiagen) using primers for the human *MAPK1, MECP2, PCDH7 or PCDHB1 *(QT00065933, QT00039361, QT01005662, or QT01019543, respectively, Qiagen), or mouse *Mecp2, Pcdh7 *or *Pcdhb1 *(QT00268555, QT01052366, or QT01055453) genes. The expression level of each gene was normalized against that of human *GAPDH *or mouse *Gapdh *(QT01192646 or QT01658692, Qiagen). All qRT-PCRs were performed in triplicate.

## Statistical Analysis

Results are given as the mean +/- SEM. The significance of differences was determined by Student's t-test for single comparisons and analysis of variance (ANOVA) for multiple comparisons.

## Authors' contributions

KM and TH performed luciferase assay, RNA interference, quantitative PCR and drafted the manuscript. MS and TT performed ChIP assay, vector construction luciferase assay. KE and KT performed bisulfate sequence. MI and YG provided experimental advice for Mecp2 null mouse and RTT patients. TN, SY and JI performed microarray analysis. TK designed the study and wrote the manuscript. All authors read and approved the final manuscript.
